# Tree age did not affect the leaf anatomical structure or ultrastructure of *Platycladus orientalis* L. (Cupressaceae)

**DOI:** 10.7717/peerj.7938

**Published:** 2019-10-29

**Authors:** Qianyi Zhou, Zhaohong Jiang, Xin Zhang, Qing Lai, Yiming Li, Fei Zhao, Zhong Zhao

**Affiliations:** 1 Key Comprehensive Laboratory of Forestry, College of Forestry, Northwest Agricultural and Forestry University, Yang Ling, Shaanxi, China; 2 College of Life Science, Northwest Agriculture and Forestry University, Yangling, Shaanxi, China; 3 Beijing Agricultural Technology Extension Station, Beijing, China

**Keywords:** Ancient tree, *Platycladus orientalis*, Anatomical structure, Ultrastructure, Chloroplast, Mitochondria, Tree aging, Tree age

## Abstract

Tree aging is a new research area and has attracted research interest in recent years. Trees show extraordinary longevity; *Platycladus orientalis* L. (Cupressaceae) has a lifespan of thousands of years. Ancient trees are precious historical heritage and scientific research materials. However, tree aging and tree senescence have different definitions and are poorly understood. Since leaves are the most sensitive organ of a tree, we studied the structural response of leaves to tree age. Experiments investigating the leaf morphological structure, anatomical structure and ultrastructure were conducted in healthy* P. orientalis* at three different ages (ancient trees >2,000 years, 200 years < middle-aged trees <500 years, young trees <50 years) at the world’s largest planted pure forest in the Mausoleum of the Yellow Emperor, Shaanxi Province, China. Interestingly, tree age did not significantly impact leaf cellular structure. Ancient *P. orientalis* trees in forests older than 2,000 years still have very strong vitality, and their leaves still maintained a perfect anatomical structure and ultrastructure. Our observations provide new evidence for the unique pattern of tree aging, especially healthy aging. Understanding the relationships between leaf structure and tree age will enhance the understanding of tree aging.

## Introduction

Aging is the last stage of plant development. However, aging has been a familiar but poorly understood issue in biology for a long time (Kirkwood, 2005; Zhou et al., 2019). Thousands of studies have been conducted to research the characteristics and causes of aging in human, animals and herbaceous plants (Guarente, 2014; Jones et al., 2014). However, tree aging is a new research area and has attracted research interest in recent years (Leopold, 1975; Jones et al., 2014). Although aging and senescence are familiar concepts, defining them is challenging. Aging and senescence are always mentioned together and sometimes used interchangeably, leading to confusion about the two words. From a language viewpoint, both of the terms “aging” and “senescence” are derived from the Latin for “growing old.” However, from a scientific viewpoint, aging and senescence have different connotations. Aging is the process of growing old and refers to individuals older than a certain boundary. The term is especially applicable to the acquisition of the physical and mental characteristics of old age. Senescence indicates organismal functional decline or physical recession of individuals (Noodén, 1988; Thomas et al., 2003; Hongbao, Young & Yan, 2014). Aging is a key life process along with germination, growth, maturation, and death and is accompanied by organ recession and decay. Senescence can occur in individuals at any age. For each individual organism, aging is divided into individual aging and cellular aging, which are distinct. However, there is not a clear relationship between individual lifespan and the longevity of organs, tissues and cells in plant species, especially trees (Skulachev, 2011; Thomas, 2013). Aging factors in plants are quite different from those in animals and other organisms (López-Otín et al., 2013) as main factors associated with animal aging do not appear to be as important in plant aging (Flanary & Kletetschka, 2005; Herbig & Sedivy, 2006).

Trees show extraordinary longevity, and some species can even live more than a thousand years (Swetnam & Brown, 1992). Tree aging was first mentioned by Westing in 1964 (Westing, 1964). The main theories in tree aging research can be divided into five aspects (Brutovská et al., 2013): the programmed death theory of aging, the damage accumulation theories, the aging theory associated with organism size, the evolutionary compromise theory and the adaptive theory of tree aging. To date, no universally agreed-upon theory has been developed by researchers. The longevity of trees strongly depends on the plant species and environmental conditions (Shulaev et al., 2008). Some extremely old plants maintain a high degree of vitality (Rajjou & Debeaujon, 2008; Thomas, 2013).

Trees have a specific growth pattern compared to humans, animals, insects and herbaceous plants (Jones et al., 2014). After an initial high level of mortality in the early stage of the tree lifespan, the mortality rate generally maintains a stable trend. During the period of tree maturation to senescence, the reproduction rate of trees increases with tree age. Although the survival rate of trees decreases with increasing tree age, some ancient trees still survive for thousands of years.

*Platycladus orientalis* L. (Cupressaceae) is a major tree species for afforestation and reforestation in northern China, especially in severe drought-prone and water and soil erosion-susceptible arid and semiarid areas such as the Loess Plateau in China (Wang et al., 2016). It is worth mentioning that *P. orientalis* has a lifespan of thousands of years. Many of these ancient trees were planted near the tombs of ancient emperors, Buddhist temples and famous national parks around the country. These trees have high historical, economic and scientific value, especially in tree aging research (Zhu & Lou, 2013). The world’s oldest *P. orientalis* is located in the Mausoleum of the Yellow Emperor in Shaanxi Province, China, which has a history of more than 4,000 years (Chang et al., 2012a; Zhang et al., 2015). However, research on the aging of these trees is rare.

To study the issue of biological aging, the most critical point is choosing an appropriate and convincing sample (Becker & Apel, 1993). Previous studies on human aging chose centenarians as experimental subjects and focused on populations with high numbers of long-lived people (Willcox, Willcox & Poon, 2010; Suzuki, Willcox & Willcox, 2016). The results of these studies are greatly influenced by various environmental and genetic factors for each individual. However, in terms of material collection, performing senescence studies on trees is far more convenient than sampling living human tissue. Therefore, to study the age-related issues of trees, the combination of the genetic origin and growing environment of the tree can describe the issue more objectively. Overall, *P. orientalis* from the Mausoleum of Emperor Huang forest shared similar genetic backgrounds, environmental and climatic conditions, with an extensive age structure, representing a valuable sampling population for tree aging-related research.

Leaf is the most sensitive organ to environmental change and plant growth (Lim, Kim & Gil Nam, 2007). Leaf senescence has a close relationship with the senescence of the whole plant (Lim, Kim & Gil Nam, 2007). However, the senescence of leaves is not equal to the senescence of trees. Unlike deciduous trees, conifers do not renew their crowns every year, and the leaves themselves are metabolized (Zhou et al., 2019). As the most important cell organelles of plant photosynthesis, chloroplasts greatly influence the senescence of trees (Doorn & Yoshimoto, 2010). Chloroplast morphology and structure differ substantially among different plant species but are relatively stable within the same organ (Veromann-Jürgenson et al., 2017). However, chloroplasts are organelles with low stability and high sensitivity. Under different environmental conditions, the chloroplast produces different structural adaptations (Lichtenthaler et al., 1981). At present, much research focuses on the chloroplast ultrastructure under different environmental conditions and stresses (Sánchez-Pardo, Fernández-Pascual & Zornoza, 2014; Shao et al., 2014; Clemente-Moreno, Hernández & Diaz-Vivancos, 2015; Bejaoui et al., 2016; Shao et al., 2016; Shah et al., 2017; Zou et al., 2017). These abiotic and biotic factors usually have a strong influence on leaf structure and function.

The anatomical structure of leaves has often been used to understand plant adaptations to environmental changes (Fahn, 1986; Bosabalidis & Kofidis, 2002; Poorter & Bongers, 2006; Tosens et al., 2012a) because of its close relationship to plant functional parameters (Rossatto & Kolb, 2009). Leaf cuticles are mainly associated with the capability of the plant to resist environmental stress, such as drought, salinity, and heat (Riederer & Schreiber, 2001). However, the most relevant ultrastructure research on plant senescence at the cellular level is mainly from crops (Mitsuya, Takeoka & Miyake, 2000; Yamane et al., 2003; Vičánková & Kutík, 2005). Chloroplast structure has a highly significant relationship with physiological function (Chin & Beevers, 1970). Moreover, the integrity of chloroplast ultrastructural integrity guaranteed the site of active photophosphorylation of the ATP enzyme and the PS II center (Kutík et al., 2000; Mitsuya, Takeoka & Miyake, 2000; Yamane et al., 2003).

Unlike typical bifacial leaves, such as those of *Sophora japonica* trees (Quy, Zhou & Zhao, 2017), the leaf branches of *P. orientalis* trees have no obvious distinction between the front and back (Hamidipour et al., 2011). *P. orientalis* leaves are considered scale leaves composed of many very small scale-like leaves clinging to small branches in a crossed arrangement. The *P. orientalis* scale leaf anatomical structure shows a unique typical symmetrical structure that can be distinguished from that of other conifer trees (Hamidipour et al., 2011). However, certain structural strategies associated with plant lifespan, such as anatomical structures and ultrastructures, remain poorly understood (Coelho et al., 2013). Research on the tree ultrastructural response to aging is lacking (Bacic et al., 2004). The anatomical structures and ultrastructures of trees at different ages have not yet been reported.

According to the special growth rate curve of tree species compared to that of animals and herbaceous plants mentioned above, we hypothesized an unidentified relationship involving the cellular anatomical structure, cellular ultrastructure and tree age. We conducted this study by characterizing the leaf anatomical structure and ultrastructure in newly grown leaves of ancient trees to recognize the relationships between the leaf structure, tree senescence and tree aging.

In this research, we studied the morphological, anatomical and ultrastructural changes in leaf tissues of *P. orientalis* at different tree ages. Our objective was to determine the leaf structural response to tree age in *P. orientalis.*

## Material and Methods

### Plant material

The study forest is an ancient *P. orientalis* forest in the Mausoleum area of the Yellow Emperor located on the Loess Plateau, Huangling County, Yan’an City, Shannxi Province, China, latitude 35°34′N, longitude 109°15′E. This area has between 1,100 and 1,300 annual hours of sunshine, a frost-free period of 170 d, an altitude range of 1,100 to 1,200 m, and a typical temperate continental climate with distinct seasonal features. The average annual temperature is 9.4 °C, and the average annual precipitation is 596.3 mm, while the average annual evaporation capacity is 487.3 mm (Zhou et al., 2019). The history of the artificial pure *P. orientalis* forest exceeds 4,000 years according to historical records and scientific research (Chang et al., 2012a; Zhang et al., 2015).

We set up three 50 m by 50 m sample plots in the forest. Samples were collected from *P. orientalis* trees in good health selected from three sample plots. In each plot, we conducted an investigation of every single *P. orientalis* tree (tree age was determined using government records *The Local County Records of Huangling County*). Tree vitality degree was assessed using the method of *The senescent degree evaluation of trees according to above-ground parts* (Zhou et al., 2019). The distance from the trunk to the sampling site were measured following the branching of the tree. After investigation, we identified three experimental groups, including three age levels (more than 2,000 years old (old age), 200–500 years old (middle age) and less than 50 years old (young age)). In each of the three plots, one tree from each experiment group was selected. The detailed information for the 9 sampled trees is listed in Table 1. For each sample tree, five repeat samples of the current year’s scale leaf were selected at a uniform time (between 10 and 12 a.m.) on the north side (sunny side) of the central crown of the tree without signs of disease or damage. Leaf samples were collected in July 2016.

**Table 1:** Basic information and group division of sampled trees.

**Basic information of sample trees**				**Site condition of sample trees**				**Healthcondition**	**Sample group division**	The height of the sampling position (m)	**The distance from the trunk to the sampling site (m)**
**Government number of ancient tree**	**Tree age(year)**	**Height(m)**	**Diameter at breast height (mm)**	**Altitude(m)**	**Slope**	**Longitude (E)**	**Latitude(N)**				
00225	>2,000	13.6	640	1,111	33°	109°15′42.5″	35°35′18.0″	Healthy	Ancient healthy tree	11.5	1.8
00221	>2,000	15.9	607	1,112	10°	109°15′42.1″	35°35′18.1″	Healthy	Ancient healthy tree	12.0	1.6
00228	>2,000	20.7	837	1,107	21°	109°15′42.9″	35°35′17.7″	Healthy	Ancient healthy tree	15.2	2.1
∖	200–500	13.7	320	1,110	37°	109°15′41.4″	35°35′15.6″	Healthy	Middle-aged healthy tree	10.8	1.5
∖	200–500	11.5	187	1,086	31°	109°15′41.3″	35°35′14.3″	Healthy	Middle-aged healthy tree	8.5	1.6
∖	200–500	10.6	562	1,092	35°	109°15′43.9″	35°35′14.4″	Healthy	Middle-aged healthy tree	8.0	1.6
∖	<50	8.6	123.5	1,121	15°	109°15′44.1″	35°35′17.2″	Healthy	Young healthy tree	6.5	0.7
∖	<50	8.1	163	1,121	15°	109°15′44.0″	35°35′17.2″	Healthy	Young healthy tree	6.0	0.5
∖	<50	6.1	56	1,112	14°	109°15′44.6″	35°35′16.2″	Healthy	Young healthy tree	4.8	0.5

### Leaf paraffin sectioning

For anatomical studies, we collected the middle part of the annual conifer leaf. Leaves were taken from trees of different ages at the same time. Samples were fixed in formalin-acetic acid-alcohol (FAA) fixing solution (5% formaldehyde, 5% acetic acid and 90% alcohol (of 70% strength)) for three days. Then, the leaves were dehydrated in a series of graded ethanol solutions, infiltrated and embedded in paraffin wax and prepared for thin sectioning (Tosens et al., 2012b). An anhydrous ethanol and tertiary butyl alcohol mixture was used in this experiment as the dehydrating agent (Gu et al., 2014). A Leica RM2245 semiautomatic rotary microtome (Leica Microsystems, Nussloch, Germany) was used to obtain transverse thin sections. All leaf sections were 8 µm thick. These paraffin sections were stained with safranin and fast green dye and permanently mounted on slides. Samples were observed, and images were taken under a UOP UB200i microscope (UOP Photoelectric Technology Company, Chongqing, China) and measured with a Tucsen image analysis system (Tucsen Photonics, Fuzhou, China). Leaf properties, such as cuticle thickness, leaf thickness, epidermis thickness, ratio of palisade parenchyma to spongy parenchyma thickness, mesophyll cell thickness, and resin cavity width, were calculated using a Tucsen image analysis system (Tucsen Photonics, Fuzhou, China).

For each group, fifty corresponding cells per group were measured.

### Transmission electron microscopy (TEM)

The current year’s (also annual) conifer leaves from sample trees were collected and cut to four mm long at their natural width. For each sampled tree, five replicates of conifer leaves were collected. A 4% glutaraldehyde fixing solution was mixed with these leaves for preservation. The preliminary fixation procedure was conducted at 4 °C by mixing leaves with a 0.1 M phosphate buffer (pH 7.3, containing 2.5% (w/v) glutaraldehyde) for 12 h. Then, after being washed several times with phosphate buffer (pH = 7.3), the postfixation of leaves occurred in a 1% solution of osmium tetroxide for 4 h at 4 °C. A graded ethanol series of 30%, 50%, 70%, 80%, 90% and 100% was used for leaf dehydration. After immersion in propylene oxide, leaves were embedded in Epon 812 resin. A Leica RM2265 Reichert microtome (Leica Microsystems, Nussloch, Germany) was used to obtain half-thin sections. These half-thin sections were checked by optical microscopy after toluidine blue staining. A Leica EM UC7 Reichert Ultratome (Leica Microsystems, Nussloch, Germany) with a diamond knife was used to cut ultrathin sections. These ultrathin sections were poststained with uranyl acetate and lead citrate (Kang et al., 1993; Ke et al., 2013). A Hitachi HT7650 transmission electron microscope (Hitachi, Tokyo, Japan) operating at 80 kV was used to observe the ultrastructure of mesophyll cells and cellular organelles such as chloroplasts and mitochondria at magnifications between 300 and 10,000 µm.

Thirty independent measurements were recorded for each leaf ultrastructural traits. Each original data point for cell organelle characteristics was repeated three times.

### Statistical analysis

The statistical analyses of anatomical observations and TEM observations were performed using IBM SPSS Statistics (version 21.0; IBM Corp., Armonk, NY, USA). One-way analysis of variance (ANOVA) and the S-N-K test were used to compare the differences among each experimental group. The confidence level of the S-N-K test was 95% (*P* ≤ 0.05). All the data are displayed as the means ± standard errors (Mean ± SD). Microscope images were grouped using Adobe Photoshop CS5 software (Adobe Systems, San Jose, CA, USA).

## Results

### Investigation of sampled *P. orientalis* trees

Here, we investigated the following information for all *P. orientalis* trees in the three plots: tree age, tree height, diameter at breast height, site condition (altitude, slope, longitude and latitude) and health condition (Zhou et al., 2019). According to the investigation results, the basic information of the nine sampled trees and their site situations are shown in Table 1.

**Figure 1:**
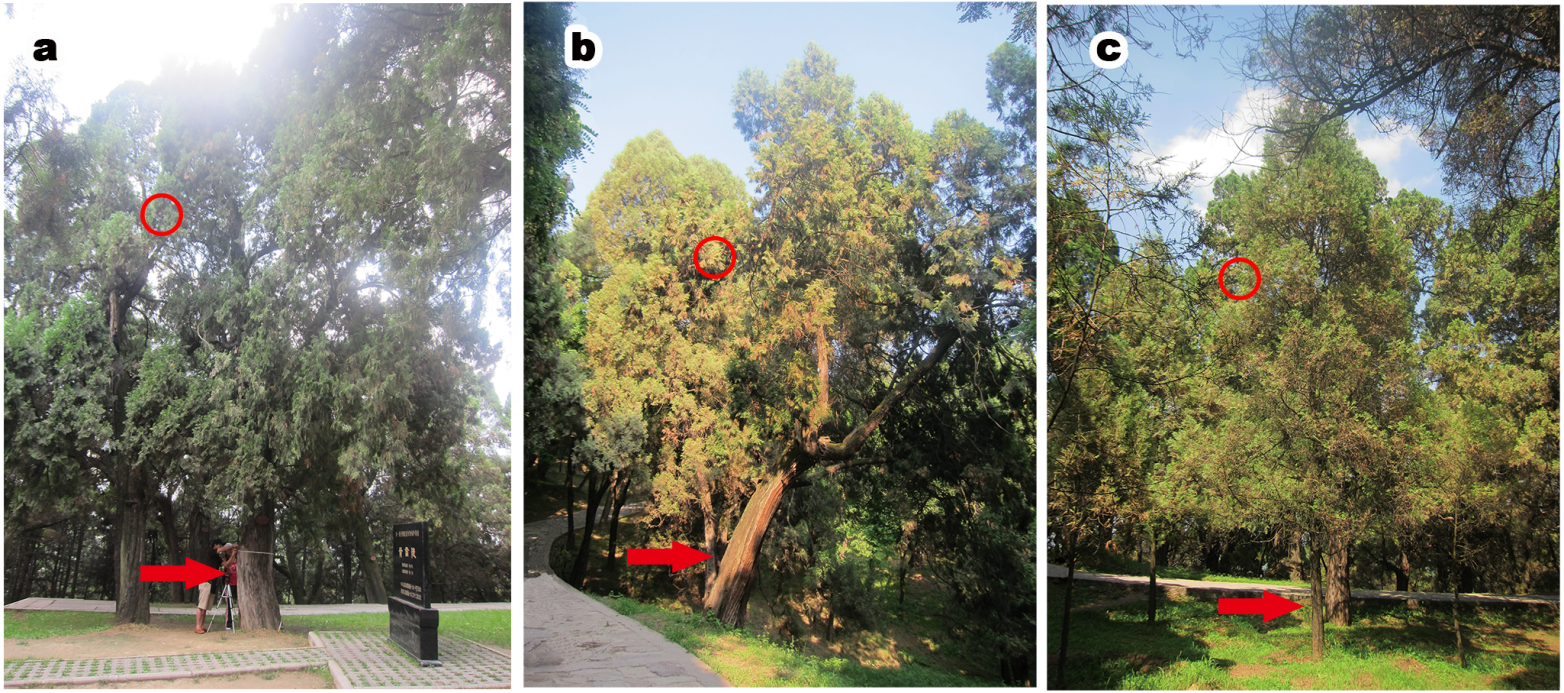
*P. orientalis* at different tree ages. (A) Ancient healthy tree; (B) middle-aged healthy tree; (C) young healthy tree. Red arrows in each images present sampled trees. Red circles in each images indicate the sampled place.

Pictures of *P. orientalis* from the three experimental groups are shown in Fig. 1. All the sampled *P. orientalis* were in good health condition. As seen from the aboveground parts and appearance, healthy *P. orientalis* leaves were green, and trees exhibited complete bark and trunks, with high vitality. The ancient tree group (Fig. 1A) was older than 2,000 years, the middle-aged tree group was aged between 200–500 years, and the young tree group was less than 50 years old. Tree height and diameter at breast height (Fig. 1, Table 1) were clearly greater in ancient trees than in middle-aged and young trees.

### Leaf anatomical structure of *P. orientalis* at different tree ages

The leaf anatomical traits of *P. orientalis* at three different tree ages are listed in Table 2. For statistical analysis of the anatomical structure, several slices with different views were observed, and seven traits of the current year’s scale leaf were measured.

**Table 2:** Anatomical properties of scale leaf of *P. orientalis* at different tree age.

Traits	Ancient tree	Middle-aged tree	Young tree
Leaf thickness (μm)	387.11 ± 46.18^b^	683.59 ± 48.34^a^	685.77 ± 22.39^a^
Cuticle thickness (μm)	5.68 ± 1.29^a^	4.01 ± 0.79^b^	3.94 ± 0.81^b^
Epidermis thickness (μm)	21.68 ± 1.78^c^	27.04 ± 5.23^a^	24.53 ± 2.66^b^
Spongy parenchyma cell thickness (μm)	36.53 ± 2.40^c^	42.76 ± 7.74^a^	39.83 ± 5.50^b^
Palisade parenchyma cell thickness (μm)	61.93 ± 6.23^c^	70.05 ± 8.34^b^	73.29 ± 8.16^a^
Ratio of palisade /spongy	1.70 ± 0.21^a^	1.69 ± 0.39^a^	1.87 ± 0.31^a^
Resin cavity width (μm)	93.60 ± 11.25^c^	175.75 ± 25.35^b^	193.78 ± 23.9^a^

**Notes.**

Data are presented as the mean ± SD (*n* = 50).

In each trait, different lowercase letters denote there are significant differences, and the same lowercase letter denote there are no significant differences among data at different tree age according to the S-N-K test at *P* < 0.05.

The ancient tree group had the lowest mean leaf thickness of 387.11 µm (Table 2). With increasing tree age, leaf thickness showed a decreasing trend. There was no significant difference in leaf thickness between middle-aged and young trees. Compared to all three groups, ancient *P. orientalis* trees exhibited the thickest cuticle. Cuticle thickness declined 1.74 µm from ancient trees to young trees. However, no significant difference in cuticle thickness was observed among the ancient, middle-aged and young tree groups. The thickness of epidermal cells (Table 2) did not decrease with tree age. The middle-aged healthy tree group had the highest epidermal thickness. Palisade parenchyma cell thickness (Table 2) was much greater than spongy parenchyma cell thickness in all three experimental groups, but the ratio of palisade/spongy cells did not show statistically significant differences among all ancient-aged, middle-aged and young-aged groups. Spongy parenchyma cell thickness was highest in middle-aged trees and lowest in ancient trees. Palisade cell thickness was highest in young trees and lowest in ancient trees. Although the difference in palisade cell thickness and spongy cell thickness was not consistent for the different tree age groups, the lowest value of the two traits was observed in the ancient tree group at the same time. There was a strong relationship between the two traits. With the increase in tree age, the resin cavity (Table 2) showed a decreasing trend.

**Figure 2:**
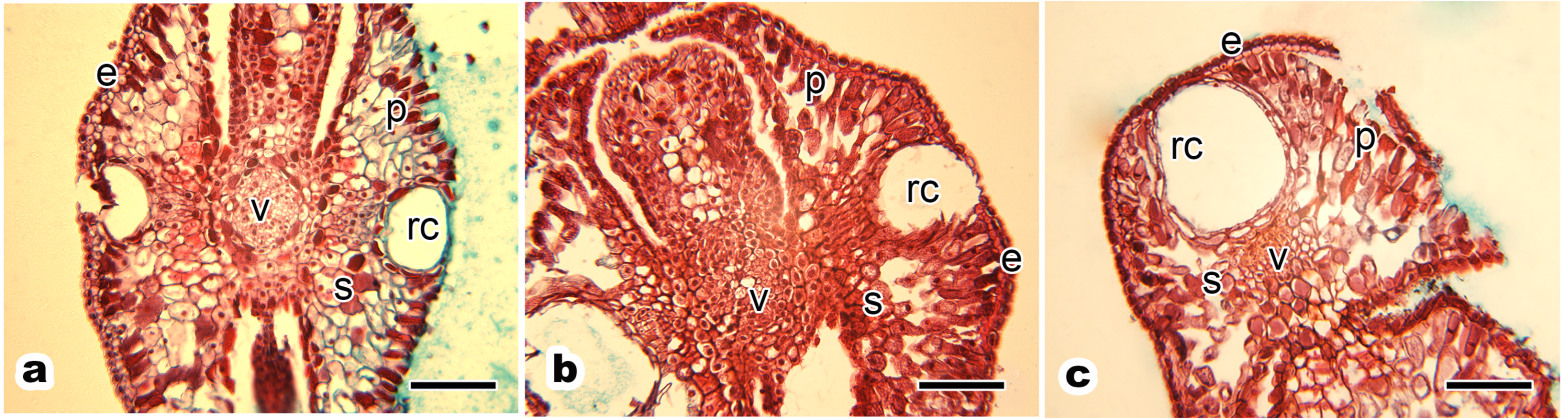
Anatomical structure of current year’s leaf of *P. orientalis* trees of different ages. (A) Ancient tree; (B) middle-aged tree; (C) young tree. Letters in images indicate the following: e, epidermis; p, palisade mesophyll cell; s, spongy mesophyll cell; v, vein; rc, resin channel. Scale bars in all images are 200 µm.

The transverse paraffin sections showed a clear view of the blade cross section. The anatomical structure of the *P. orientalis* tree had no obvious distinction between the front and back, with resin gland grooves on the middle of the leaf blade oriented axially (Fig. 2).

The leaf anatomical structure of *P. orientalis* at different tree ages (ancient, middle-aged, young) showed no significant changes with tree age. Current year’s leaves of healthy ancient (Fig. 2A), middle-aged (Fig. 2B) and young (Fig. 2C) *P. orientalis* trees had a perfect anatomical structure with typical symmetry. The upper and lower epidermis were composed of a single layer of small epidermal cells. The central vein of the leaf consisted of closely arranged vein cells. The resin channel occurred on both sides of the veins with a smooth edge. The mesophyll cells of scale leaves were composed of spongy tissue and palisade tissue, which were spaced closely.

### Leaf ultrastructure of *P. orientalis* of different tree ages

In this study, the cell wall thickness, the size and shape of chloroplasts and mitochondria and the starch in chloroplasts of *P. orientalis* at different tree ages were observed using TEM. The detailed chloroplast, mitochondria and starch traits are presented in Table 3. Thirty independent mesophyll cells were measured for each trait.

**Table 3:** Ultrastructure properties of mesophyll cells of *P. orientalis* with different tree ages.

Traits	Ancient tree	Middle-aged tree	Young tree
Cell wall thickness (μm)	0.40 ± 0.11^b^	0.46 ± 0.05^b^	0.55 ± 0.06^a^
Chloroplast length (μm)	3.99 ± 0.72^b^	4.49 ± 0.72^a^	3.60 ± 0.45^b^
Chloroplast width (μm)	2.29 ± 0.63^a^	2.50 ± 0.65^a^	2.38 ± 0.32^a^
Chloroplast number/cell cross section	5.3 ± 1.25^a^	5.8 ± 0.42^a^	5.6 ± 1.43^a^
Mitochondria length (μm)	0.97 ± 0.60^a^	0.78 ± 0.22^a^	0.69 ± 0.12^a^
Mitochondria width (μm)	0.68 ± 0.32^a^	0.58 ± 0.14^a^	0.48 ± 0.08^a^
Mitochondria number/cell cross section	10.1 ± 2.08^a^	8.5 ± 1.43^b^	6.8 ± 1.69^c^
Starch grain length (μm)	2.00 ± 0.62^b^	2.85 ± 0.66^a^	0.51 ± 0.24^c^
Starch grain width (μm)	1.10 ± 0.53^b^	1.65 ± 0.67^a^	0.36 ± 0.20^c^
Starch grain number/chloroplast	1.8 ± 0.42^a^	1.6 ± 0.70^a^	1.3 ± 0.48^a^

**Notes.**

Data are presented as the mean ± SD (*n* = 30).

In each trait, different lowercase letters denote there are significant differences, and the same lowercase letter denote there are no significant differences among data at different tree age according to the S-N-K test at *P* < 0.05.

There is no significant differences of the cell wall thickness between ancient tree group and middle-aged tree group. Young trees had the thickest cell walls of all groups (0.55 µm) (Table 3). The length of chloroplasts at different ages ranged from 3.60 to 4.49 µm, whereas the width of chloroplasts ranged from 2.29 to 2.50 µm. The largest chloroplast length and width were observed in the middle-aged healthy tree group (4.44 µm and 2.36 µm, respectively). The chloroplast width was not significantly different among the three groups of healthy trees. Among the three tree age groups, chloroplast length showed a significant difference, but the number of chloroplasts per cell cross section was almost the same. The mitochondrial traits are presented in Table 3. Mesophyll cells of *P. orientalis* generally had five to ten mitochondria. The highest number of mitochondria per cell cross section was 10.1 for ancient healthy trees. The smallest number of mitochondria per cell was 6.8 for young healthy trees. The numbers of mitochondria per cell cross section decreased with tree age in three tree groups. The length and width of mitochondria showed no significant differences among all three experimental tree groups. Starch is a product of photosynthate accumulation in chloroplasts. As shown in Table 3, the length of starch grains of middle-aged trees was slightly higher than that in ancient and young trees. The length and width of starch grains first increased and then decreased from ancient to young age. The number of starch grains showed no difference among tree ages with ANOVA.

**Figure 3:**
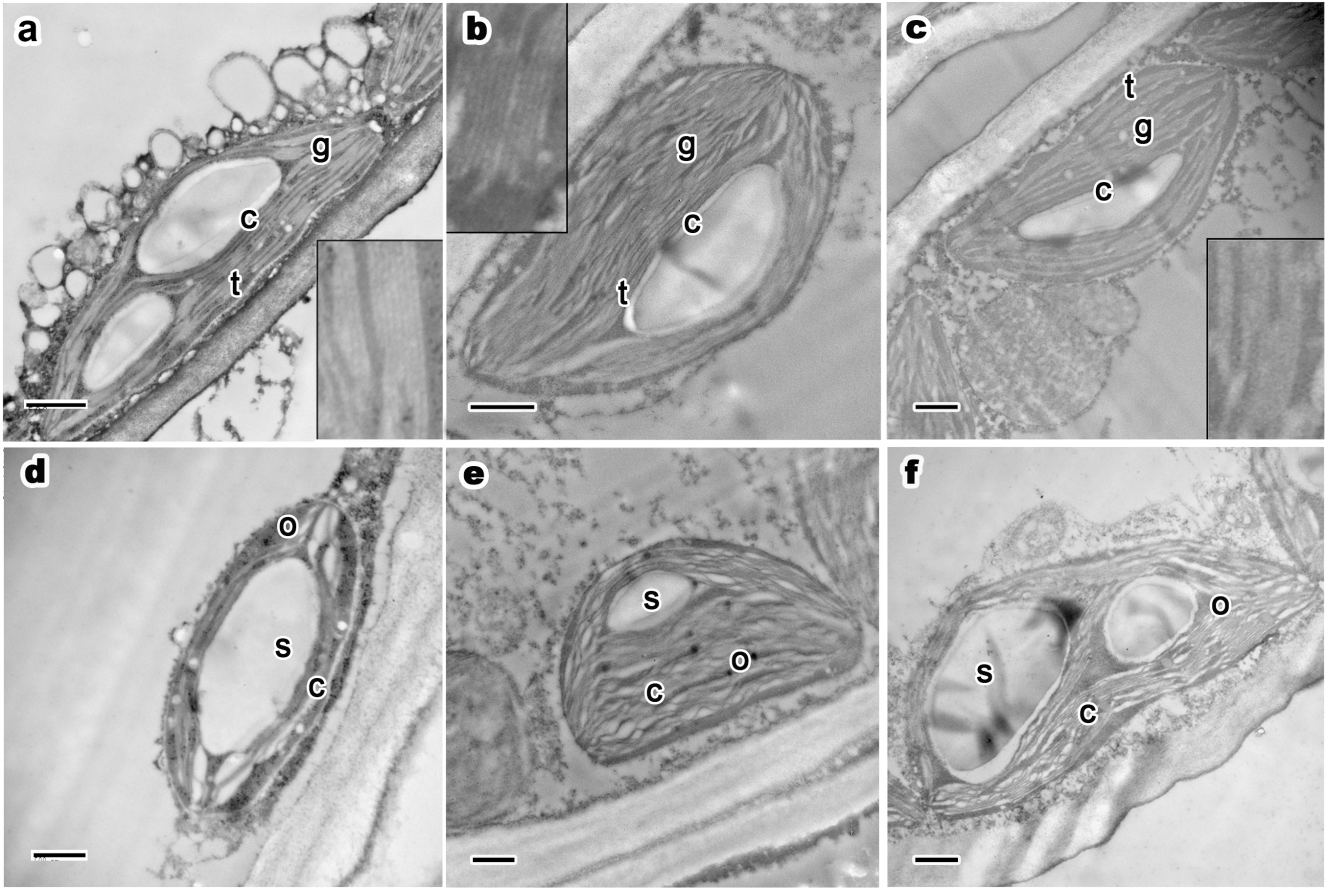
Ultrastructure of current year’s leaf’s chloroplast, osmiophilic granules and starch grains of *P. orientalis* tree at different tree ages. (A & D), Ancient tree; (B & E), middle-aged tree; (C & F), young tree. Letters within images represent the following: c, chloroplast; t, thylakoid; g, grana lamellae; s, starch grain; o, osmiophilic granule. Image on black rectangle in picture (A), (B) and (C) corresponds to enlarge figure of chloroplast grana lamellae and thylakoid of each experimental group. Scale bars in all images are 500 nm.

As the most important organelle of plant mesophyll cells, chloroplast ultrastructure did not change apparently with tree age. *P. orientalis* in the three tree groups generally had an intact, smooth, double-layered chloroplast membrane structure (Figs. 3A–3C). The thylakoids had clear, thick stacks composed mostly of chloroplast lumen (images in black rectangles in Figs. 3A–3C). Low-layered grana lamellae represented only a very small part of the interspace and often appeared as a connection between thick thylakoid stacks. The dark, compact chloroplast stroma showed that chloroplasts of healthy trees have high electron density, which promote the rapid rate of photosynthesis.

Osmiophilic granules are formed by the combination of lipid droplets, proesters, ketones and osmium tetroxide during the postfixation process of TEM sample fixation. These small stroma-distributed particles in chloroplasts generally remained the same when the trees were in a good state of health, even when the trees were older than two thousand years. In healthy mesophyll cell chloroplasts of ancient (Fig. 3D), middle-aged (Fig. 3E), and young trees (Fig. 3F), osmiophilic granules were small and evenly distributed in the chloroplast stroma. Moreover, for trees at different ages but the same state of health, no obvious differences were observed with respect to the size, number or distribution of osmiophilic granules.

Most chloroplasts had apparent starch grains. Starch grains in chloroplasts basically maintained the same traits (as shown in Table 3). Most chloroplasts in the three experimental groups had one to two starch grains per chloroplast. The number of starch grains is small but the volume is huge in summer. In all groups, starch grains in chloroplasts were distributed in the space between grana stacks. There were no apparent differences among the groups. In ancient healthy (Fig. 3D), middle-aged healthy (Fig. 3E), and young healthy tree (Fig. 3F) groups, chloroplasts and thylakoid stacks were intact. The presence of starch grains did not affect the normal ultrastructure and integrity of chloroplasts. In summary, no differences were observed among different tree age groups.

**Figure 4:**
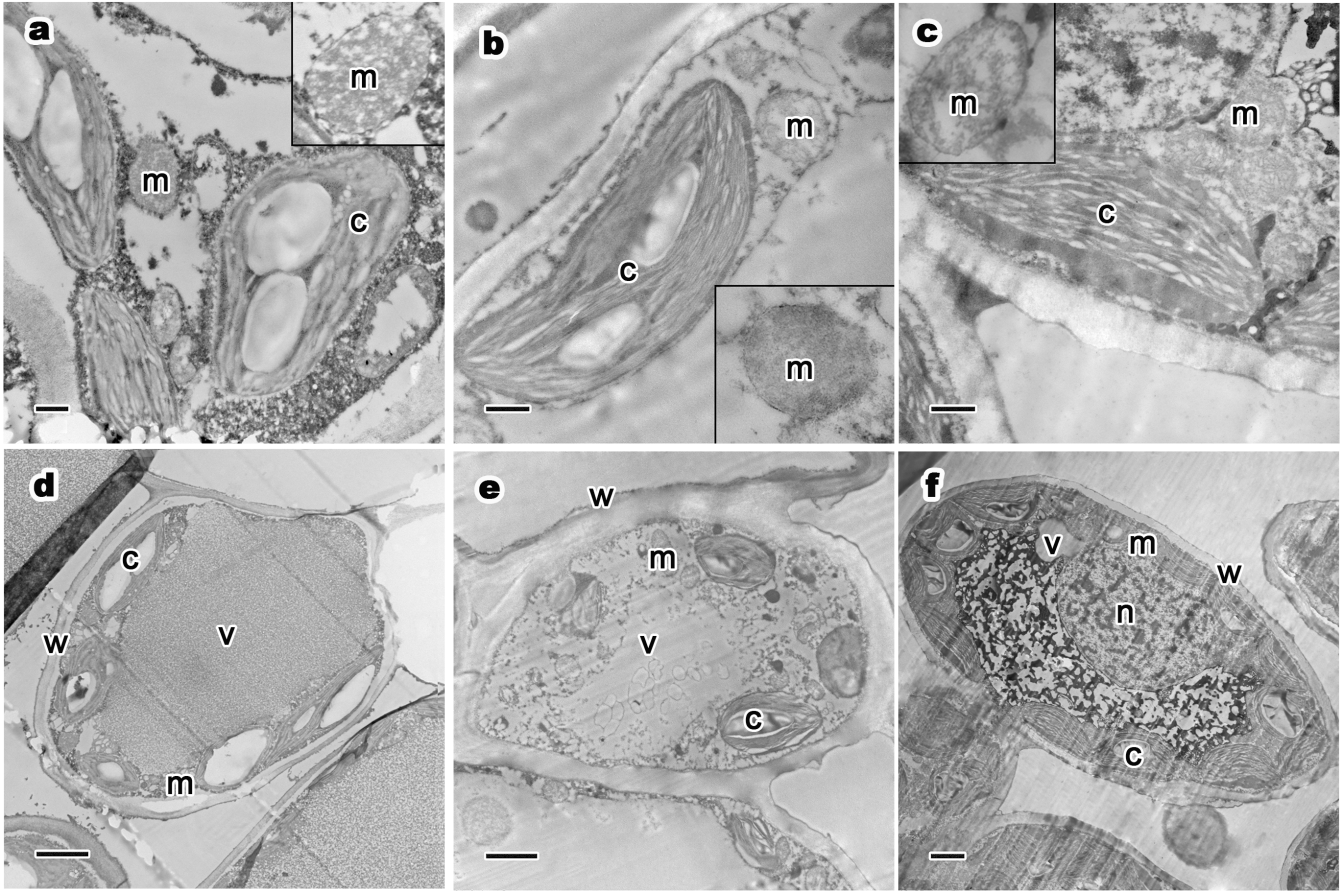
Relative distribution between mitochondria and chloroplasts, and ultrastructure of mesophyll cells and organelles of *P. orientalis* tree at different tree ages. (A & D) Ancient tree; (B & E) middle-aged tree; (C & F) young tree . Letters in images represent the following: c, chloroplast; m, mitochondria; w, cell wall; v, vacuole; n, nuclear. Image on black rectangle in picture (A), (B) and (C) corresponds to enlarge figure of mitochondria of each experimental group. Scale bars in image (A), (B) and (C) are 500 nm and in (D), (E) and (F) are 2 µm.

The relative distribution of mitochondria and chloroplasts also looked the same among cells from trees of different ages. In ancient trees (Fig. 4A), mitochondria had abundant cristae and were arranged very close to the chloroplasts. With different aged healthy trees (Figs. 4A–4C), the structural integrity of mitochondria did not change notably. Mitochondria were sharp-edged and intact and had a high density of mitochondrial cristae. All mitochondria maintained round or oval shapes and showed no significant changes in cristae. The results of the observations showed that tree age did not have an impact on the relative distribution of mitochondria and chloroplasts.

The ultrastructure of mesophyll cells and organelles of *P. orientalis* under different tree ages are shown in Figs. 4D–4F. The main organelles analyzed in this part of the study included the cell wall, chloroplast, mitochondria and vacuole.

Cell wall thickness slightly decreased with tree age (Table 3 and Figs. 4D–4F). Moreover, the degree of bending of the cell wall did not increase with increasing tree age. The chloroplasts in the mesophyll cells of healthy but different aged *P. orientalis* trees were located at the cell edge, close to the cell plasma membrane and cell wall ( Figs. 4D–4F). Most chloroplasts were long rods and bicuspid shaped. The changes in mitochondria distribution (Fig. 4) and traits such as number and shape (Table 3) are described above.

The vacuoles of mesophyll cells in healthy ancient, middle-aged and young trees were large, centrally distributed and indivisibly, singly monolithic.

Interestingly, the leaf ultrastructure exhibited no significant changes between trees of the different tree ages. Tree age did not significantly impact leaf cellular structure.

## Discussion

In this study, we detected the following main features in the current year’s leaves from trees of varying ages: (a) anatomical traits and anatomical structure of the leaf tissue; (b) ultrastructural properties and cellular ultrastructure of mesophyll cells. As the most vital organelles of mesophyll cells, the leaf chloroplast structure and mitochondrial structure of *P. orientalis* had no relationship with tree age according to our research. Combining observations of leaf morphological features, anatomical structures and ultrastructures in trees of different ages, little difference was found among the different tree age groups.

The leaf is one of the largest organs in trees that is exposed to the environment (Lim, Kim & Gil Nam, 2007). During the life of a tree, it is vulnerable to environmental factors such as altitude, temperature, rainfall, and soil conditions and nutrients. The internal physiology and gene expression are also affected. These factors can impart regular and apparent morphological and structural changes to the leaves of trees. The structural response can provide an important basis for tree aging and can also be used to quickly evaluate the overall recession of ancient trees, which can be improved by timely and effective rejuvenation methods.

The aging of whole trees is completely different from leaf senescence (Lim, Kim & Gil Nam, 2007). This phenomenon was especially significant for deciduous trees such as *Sophora japonica* (Quy, Zhou & Zhao, 2017). Unlike deciduous trees, the canopy of evergreen trees (such as *P. orientalis*) does not disappear and become renewed every year, but its leaves still undergo metabolism. Because leaves from one and two years in the past cannot reflect whole tree senescence because of their own structural changes, well-developed current year’s leaves with intact anatomical structures are the best choice to study the leaf structural response to tree aging (Gepstein, 2004).

Tree cellular structure is greatly affected by tree health. The health status of a tree determines the morphology of the whole tree. Leaves are a vital organ reflecting whole-tree senescence (Lim, Kim & Gil Nam, 2007). Trees in poor health showed a visible decline in appearance, including dead branches, a lower density of leaves, yellow foliage, damaged bark and poor growth potential (Zhou et al., 2019).

The anatomical structures showed no response to tree aging. The classic anatomical structural characteristics of a healthy *P. orientalis* leaf include thick cuticles, appropriate epidermal thickness, a smooth resin cavity, proper leaf thickness and regular spongy and palisade parenchyma cells. The cuticle thickness is closely related to the relative water content of leaves. The thick cuticle helps plants withstand drought and other abiotic stresses (Yao, Gao & Cheng, 2001). Moreover, the plant epidermis is a multifunctional tissue that plays important roles in reproduction, defense and pollinator attraction (Glover, 2000). Leaf cuticles are mainly associated with the capability of plants to resist environmental stress, such as drought, salinity, and heat (Riederer & Schreiber, 2001). The ancient *P. orientalis* had thick cuticles and an appropriate epidermal thickness, which may indicate good environmental resistance of the whole tree. The leaf resin canal is a mature leaf organizational structure, and the resin acids help trees to resist diseases, pests and fungi (Franich, Gadgil & Shain, 1983; Rocchini, Lindgren & Bennett, 2010). However, the wide and disordered structure of leaf resin channels seriously affected the number and distribution of leaf mesophyll cells (spongy and palisade parenchyma cells), which could lead to a decline in the photosynthetic ability of trees (Lawson et al., 2003). These changes fully demonstrate that the physiological function of trees is closely related to their anatomical structure. Similar results were obtained for anatomical changes in *Citrus* trees with boron toxicity (Huang et al., 2014), for which a significant change was observed in leaf cortex cells and phloem tissues. The spongy parenchyma cells and palisade parenchyma cells were void and distorted (Huang et al., 2014). The ratio of palisade/spongy cells determined the shape and size of leaf mesophyll cells and showed no significant difference among trees of different ages. Thus, the mesophyll cell itself did not undergo momentous shape changes.

The leaf ultrastructure also showed no response to tree age. Trees of all three age groups (including ancient trees, middle-aged trees and young trees) had sharp-edged mitochondria, thickly stacked thylakoids in crescent-shaped intact chloroplasts, a large central vacuole, a large number of chloroplasts, fairly close chloroplasts and mitochondria and regular cell walls. Chloroplasts are the most sensitive organelles within mesophyll cells. When plants reach senescence, chloroplasts degrade and dismantle before other organelles, leading to a decreased photosynthetic rate (Hörtensteiner & Feller, 2002; Saco, Martin & San Jose, 2013). In our experiment, chloroplasts, mitochondria, and other important organelles in mesophyll cells showed no relationship with tree age, which agreed with Hörtensteiner and Feller’s experiment, in which mitochondria remained functional during plant senescence (Hörtensteiner & Feller, 2002). Mitochondria have a close relationship with senescence according to the free radical theory (Shigenaga, Hagen & Ames, 1994). Reactive oxygen species (ROS) contribute to senescence in both animals and plants (Trifunovic et al., 2004; Guo & Crawford, 2005; Noctor, De Paepe & Foyer, 2007). ROS generated in cells arise in a variety of ways. Among all oxidative burdens from several cellular sources, mitochondria take on the vast majority of the cellular ROS burden (estimated at approximately 90%) (Turrens, 2003). In senescent plants, mitochondria accumulated large amounts of ROS, which might damage mitochondrial cristae, decrease the mitochondrial energy production rate and the metabolism of the mesophyll cell, leaf tissue, or the whole plant (Nemoto et al., 2000). Most current research on mitochondrial ultrastructural and functional changes has been concentrated in algae, crops and model plants such as *Arabidopsis thaliana* (Romo-Parada et al., 1991; Xu et al., 2013; Romanova et al., 2016; Fanello, Bartoli & Guiamet, 2017). Woody plants such as *P. orientalis* are completely unstudied in this area. Therefore, the observations from our study, including those of chloroplasts, thylakoids, the relative distribution between chloroplasts and mitochondria, starch grains, osmiophilic granules and cell walls, provide new evidence of the unique pattern of tree aging, especially healthy aging.

Tree age did not significantly impact leaf cellular structure. Trees from ancient, middle-aged and young age groups had a similar appearance at the same health status or degree of senescence. For example, ancient healthy trees, middle-aged healthy trees and young healthy trees all had perfect anatomical structures and ultrastructures despite an age difference of more than 2,000 years. Although there are no data for leaf structural (including morphological, anatomical structural and ultrastructural) responses to tree aging, research on ancient trees has been reported in recent years (Ermei et al., 2011; Wen et al., 2011; Chang et al., 2012b; Zhang et al., 2015). In *P. orientalis* seeds from different aged trees, no significant correlation was found among the germination index, MDA content and antioxidant enzymes (SOD, CAT, POD, APX and GR) activities with tree age. Moreover, the ROS content did not increase with tree age. In 3,000-year-old and 20-year-old *P. orientalis*, seeds all maintained a high germination index, active antioxidant enzyme activities, and a low content of ROS and MDA (Chang et al., 2012b). In addition, the leaves of ancient trees were in good condition (Ermei et al., 2011). In our study, all ancient, middle-aged and young trees maintained perfect leaf tissue and an intact cellular structure as long as they were in good health. With increased tree senescence, leaf structure traits including the chloroplast number, mitochondria number, vacuole size and cell wall thickness of mesophyll cells were negatively correlated with the degree of senescence (Zhou et al., 2019). This perhaps explains why some trees can live so long, even thousands of years, and still maintain good growth momentum, as though they were immortal.

In contrast to humans, animals, insects and herbaceous plants, trees had specific growth curves (Jones et al., 2014). The reproduction rate of trees increased with age and maintained a balance after maturation until the end of the lifespan. The survival rate of trees decreased with age, but some ancient trees survived after thousands of years (Jones et al., 2014).

Trees are perennial flowering plants. During the growth process of a single tree, the growth curve presented three unique states (Westing, 1964; Issartel & Coiffard, 2011; Jones et al., 2014). In the juvenile stage, the growth rate increased after germination until the mature stage. During this period, trees grow fast to become taller, stronger and sexually mature. This stage will last two to tens of years depending on the tree species. In the mature stage, trees maintain strong vitality, constantly producing large amounts of fruits, rapidly metabolizing and constantly renewing roots and leaves, and the growth rate remains relatively stable at a quite high level. This period can last more than one hundred years, even thousands of years (Lanner, 2002). That’s why trees that are more than thousands of years old can still maintain good leaf structure. For the senescent stage, trees abandon their vigorous growth in favor of a decreased growth rate, with the trunk becoming hollow and rotting, leaves turning green to yellow, fruit and seeds showing decreased vitality. This period is very fast compared to the whole lifespan of trees, with usually just a few years or less required to reach complete death (Thomas, 2013; Zhou et al., 2019).

Currently, the majority of ancient *P. orientalis* in the forest of the Mausoleum of Emperor Huang, Huangling County, Yan’an City, Shaanxi Province, China, are in a suboptimal state of health. Very few of the ancient trees are completely healthy, but dying trees also represent only a minority. Thus, timely and effective rejuvenation and protection is necessary before most ancient trees in this forest undergo irreversible deterioration. As these ancient trees represent a precious historical heritage with both scientific research and cultural value, improving their growth potential and retaining their germplasm resources is urgent.

Aging is a process, while senescence is a phenomenon affecting individuals. Senescence is the final external performance of a tree determined by complex ecological environmental impacts and internal causes over long periods. Factors leading to tree senescence include tree size, environment stress, physiological function, biochemical metabolism and gene regulation (Flanary & Kletetschka, 2005; Mencuccini et al., 2005; Brutovská et al., 2013). Examination of structural traits is only the first step of the study of senescence, and additional research will involve additional experiments (including environmental, physiological, biochemical, protein-based and genetic studies). At present, it is difficult to perform comprehensive sampling of ancient trees (for example, root sampling was not allowed by the government, while the pith was empty or partly empty due to thousands of years of erosion). The accurate age of ancient trees is difficult to determine internationally without official literature records. Sampling and analysis is difficult because of specificity. Some regions of aging trees lack data. For these reasons, current research on tree aging is rare.

The relationship between tree aging and tree leaf structure can enhance the understanding of the definition of tree aging. Based on the results of our study, the senescence or recession of trees is not entirely determined by tree age. The observation of leaf structure can indicate tree vitality in advance without damage to the precious ancient tree (Zhou et al., 2019). Ancient *P. orientalis* in forests older than 2,000 years still have very strong vitality. Our observations provide new evidence of tree aging to support the unique growth and aging model of trees (Issartel & Coiffard, 2011; Jones et al., 2014).

In this study, we examined the leaf structural response of *P. orientalis* (including morphological structure, anatomical structure and ultrastructure) to tree aging. The results revealed the relationships among leaf structure and tree age. The leaf anatomical structure and ultrastructure of *P. orientalis* had no relationship with tree age. In future studies, the underlying mechanism of the structural response to tree age and tree aging will be explored.

## Conclusion

Observations showed that tree age did not significantly impact the leaf anatomical structure or ultrastructure. Ancient *P. orientalis* in forests older than 2,000 years still have very strong vitality, and their leaves still maintained perfect anatomical structures and ultrastructures. Our observations provide new evidence of the unique pattern of tree aging, especially healthy aging.

## Additional Information and Declarations

### Data Availability

The following information was supplied regarding data availability: The raw data is available at Figshare: Zhou, QianYi (2019): Raw data of leaf structure of tree aging. figshare. Dataset. https://doi.org/10.6084/m9.figshare.8910806.v1.
